# Assessing executive functions in free-roaming 2- to 3-year-olds

**DOI:** 10.3389/fpsyg.2023.1210109

**Published:** 2023-06-29

**Authors:** Lisanne Schröer, Richard P. Cooper, Denis Mareschal

**Affiliations:** ^1^Centre for Brain and Cognitive Development, Birkbeck, University of London, London, United Kingdom; ^2^Centre for Cognition, Computation and Modelling, Birkbeck, University of London, London, United Kingdom

**Keywords:** executive function (EF) skills, children, development, naturalistic, planning

## Abstract

**Introduction:**

Core aspects of executive functions (EFs) are known to be related to academic skills such as literacy and numeracy. However, school outcomes may also be related to higher-level functions such as planning. Nevertheless, few studies have considered assessing natural manifestations of higher-level EFs in children who are on the cusp of entering formal schooling. One reason for this is the difficulty of obtaining ecologically valid measures of EFs in preschool-aged children.

**Method:**

We describe a novel task - building a striped Duplo tower subject to two constraints - designed to assess planning in real-world multi-action situation. Children were instructed to build a tower to a certain height by alternating between two different colors of blocks.

**Results:**

Performance on one of the constraints in this task was found to vary with age. Importantly, distinct components of multiple constraints planning performance predicted laboratory-based measures of inhibitory control and working memory efficacy.

**Discussion:**

Thus, this task provides a simple, cheap and effective way of assessing executive function in toddlers through the observation of natural behavior. It also opens up possibilities to investigate the neurodevelopment of EF in the real world.

## Naturalistic planning in free-roaming toddlers under 3 years of age

Executive functions (EFs) are the cognitive processes that control and regulate goal-directed behaviors (Barkley, 2012; Miyake and Friedman, 2012; Diamond, 2013). Executive functions have been investigated at a basic, core-process, level and at a higher cognitive-control level. For example, working memory, inhibition, and set-shifting are generally considered to be core components of executive functions (Miyake et al., 2000; Diamond, 2013). *Working memory* is the ability to hold information in mind for a short period of time and, if necessary, to mentally manipulate this information (Miyake and Friedman, 2012; Diamond, 2013). Evidence suggests that this ability to keep representations in mind develops in the first 6 months of life (Pelphrey et al., 2004; Reznick et al., 2004) and improves across preschool years (Gathercole, 1998, 1999; Espy and Bull, 2005). *Inhibition* is the ability to control one's attention, thoughts, behaviors, and/or emotions to override an external temptation or a strong internal predisposition (Miyake and Friedman, 2012; Diamond, 2013). Response inhibition, the ability to suppress a dominant response, particularly develops in the 1st year of life, continuing to improve over toddlerhood and the preschool years (Kochanska and Aksan, 1995; Garon et al., 2008; Kochanska et al., 2008; Friedman et al., 2011). Lastly, *set-shifting* is the ability to shift attention between different tasks and mental sets (Miyake and Friedman, 2012; Diamond, 2013); in addition, both response shifting and attention shifting develop over preschool years (Zelazo et al., 1996, 2003; Espy et al., 1999; Garon et al., 2008).

**Figure 1:**
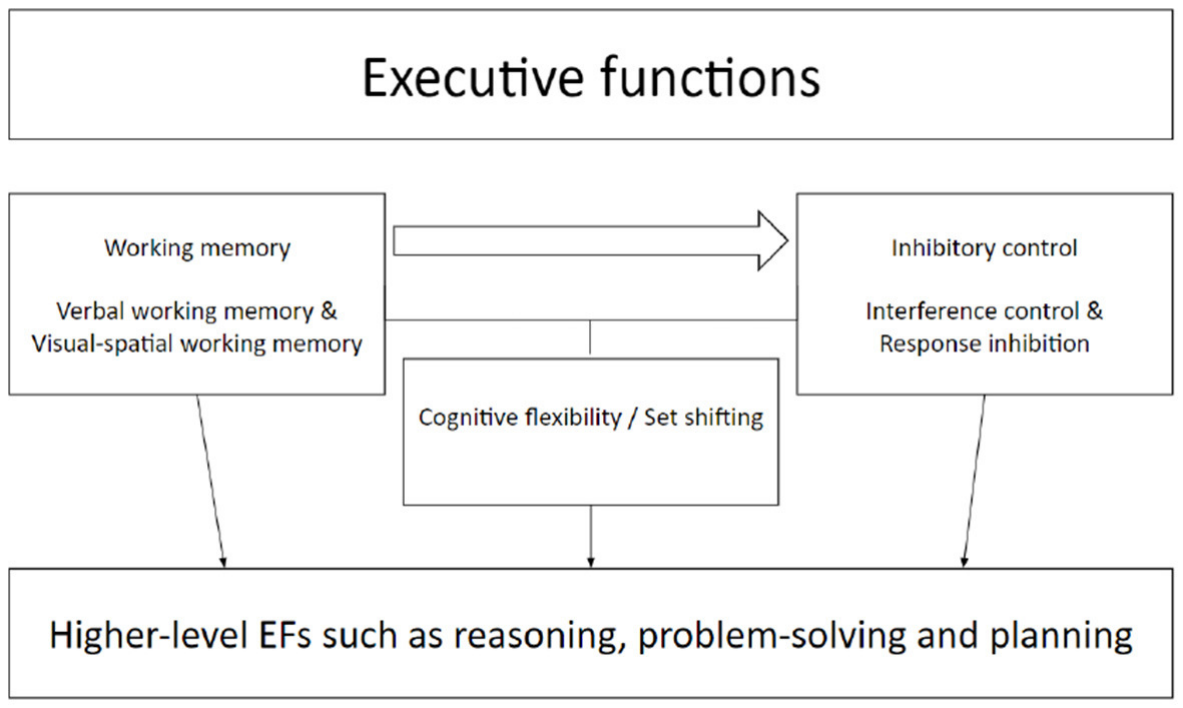
Relations between lower- and higher-level aspects of executive functions. [Adapted from Diamond (2013)].

It is hypothesized that the foundation of these core components lies in infancy or toddlerhood (Garon et al., 2008; Anderson and Reidy, 2012). However, there are also higher-level and more complex EF skills such as planning, reasoning, and problem-solving. It has been suggested that these higher EF skills are likely to be dependent on lower-level core aspects of EF (including working memory, inhibition, and set-shifting) (Figure 1, McCormack and Atance, 2011; Miyake and Friedman, 2012; Diamond, 2013). Developmental research into EF has largely been focused on the development of these lower-level core aspects of EF; however, research into the development of higher-level EF aspects has been relatively neglected. In the current study, we focus on the development of a naturalistic measurement of the higher-level EF component of *planning*. Planning is a complex set of mental and behavioral operations that brings together cognitive, emotional, and motivational resources to achieve the desired goals (Shallice, 1982; Friedman and Schonick, 2014). In past studies, planning of simple actions and action sequences in infancy and childhood has been linked to improvements in the core aspects of executive functions (Pennequin et al., 2010; Gottwald et al., 2016; Yanaoka and Saito, 2019, 2020; Schröer et al., 2021), and planning abilities on standard tasks such as the Tower of London have argued to be related to inhibition and set-shifting (Baughman and Cooper, 2007; Cooper and Marsh, 2016), suggesting a tight link between lower- and higher-level components of EF.

There is substantial evidence that the core aspects of EF are related to school readiness and academic achievement in early childhood years (e.g., Bull and Scerif, 2001; Blair and Raza, 2007; McClelland et al., 2007; Espy et al., 2010; Welsh et al., 2010; Fuhs et al., 2014; Blair et al., 2015; Blair, 2016). For example, Fitzpatrick and Pagani (2012) found that improved working memory skills in toddlerhood were related to better classroom engagement, number knowledge, and receptive vocabulary in kindergarten, while Welsh et al. (2010) found that working memory at the beginning of kindergarten was related to improved emergent literacy and numeracy skills. Furthermore, working memory efficacy has been found to contribute to variance in school achievement at the end of Grade 1 (Monette et al., 2011). Better working memory scores have also been found to provide children with a head start in mathematics and reading achievement (Bull et al., 2008). However, while both working memory and inhibition have been found to predict early arithmetic competence in children between 2 and 5 years of age, Espy et al. (2010) found that only inhibition explained unique variance in mathematical skills after controlling for other core aspects of EF. Moreover, Blair and Raza (2007) found that inhibition was positively related to mathematical ability and knowledge of letters within emerging literacy in 3- to 5-year-olds.

Similarly, many studies have found a combination of several core aspects of EF that are related to school readiness and academic achievement, suggesting that those higher EFs that are dependent on multiple core aspects of EFs might also relate to school readiness and school success. For example, the combination of inhibition and set-shifting was found by Shaul and Schwartz (2013) to be related to emergent literacy and mathematical knowledge in preschool children, while Bull and Scerif (2001) found that mathematical abilities in children of 7 years of age were associated with measures of set-shifting, inhibition, and working memory. Furthermore, the head-to-toes task, which assesses inhibition, attention, and working memory, significantly predicted emergent literacy, vocabulary, and math skills (McClelland et al., 2007). Similarly, set-shifting and inhibition at age 4 were related to mathematical achievements at age 6 (Clark et al., 2010). Lastly, low-level EF components such as improved working memory are related to more appropriate classroom behavior, such as a larger attention span, lower levels of distractibility, fewer problems in monitoring the quality of working, and better ability to generate new solutions (Gathercole et al., 2008), suggesting that EFs contribute to school success.

While it is well-known that the development of lower-level EF skills is related to improved school readiness in later life, much less is known about how the development of higher-level EF skills such as planning is related to school readiness and school success. This stands in contrast to the fact that, in later life, for example in late adolescence, higher-level EFs such as planning are known to be important for academic success (Baars et al., 2015). Furthermore, problems in classroom behavior that are associated with poor working memory are also associated with lower scores on measures of planning (St. Clair-Thompson, 2011). Only one study has shed some light on the relationship between planning and school readiness; in the study, performance on the Tower of London task during 4 to 5 years of age was predictive of early math abilities (Bull et al., 2008), but how planning in very early childhood is related to later school success remains unknown. This is an important question because the first formal school systems begin during 3 to 4 years of age in many countries. However, most developmental studies exploring EFs and school readiness focus on children 4 to 5 years of age (e.g., Bull et al., 2008; Clark et al., 2010; Welsh et al., 2010; Monette et al., 2011).

There are various tasks to provide measures of the core aspects of executive functions in early childhood (e.g., Garon et al., 2008; Howard and Melhuish, 2017). However, naturalistic tasks that can be used in everyday nursery contexts are rare. This is particularly true for children in their 3rd year of life when school readiness is of critical concern. The main reason for this is that 2- to 3-year-olds are an especially difficult age group to study as they are highly mobile and have low concentration, making them difficult to assess using rigid standardized tasks. One exception is Schröer et al. (2022) coin sorting task, which is designed to measure the ability of 2- to 3-year-olds to plan extended alternating action sequences. This task is especially simple and only requires some plastic coins and two boxes. The experimenter demonstrates sorting the coins between the two boxes in a left–right–left–right (alternating) pattern. Toddlers are then instructed to continue putting the coins in the boxes in this alternating way. Schröer et al. (2022) found that the ability to plan and execute these alternating action sequences improved between 2 and 3 years of age and, critically, was predicted by working memory capacity.

However, real-world action planning often involves multiple goals, constraints, or goal hierarchies. For example, something as simple as making a cup of tea in the morning involves a goal hierarchy of setting an overarching goal and then establishing the necessary subgoals to accomplish the task (Cooper et al., 2014). Freier et al. (2017) used a coloring task in which children had to color six farm animals following the direction of an arrow and use each coloring pencil equally often as a measure of hierarchical planning abilities in 3- to 5-year-olds. In this study, action planning involved taking into account a higher- and a lower-level goal simultaneously. While this task revealed improvements in action-planning abilities between 3 to 5 years of age (Freier et al., 2017) and could be used within a school environment, it is very difficult for 2- to 3-year-olds. Hence, our current task uses Duplo blocks with simpler instructions to investigate planning development in 2- to 3-year-olds. Toddlers were instructed to build a tall tower with a height constraint and to make it striped by alternating between two colors. Performance on these two constraints was used to assess planning improvements. This simple task is short, does not require complex materials, and is not dependent on verbal expressive abilities that are known to be poor in this age group (Colson and Dworkin, 1997).

The aim of the current study was to develop a measure of planning based on a naturally occurring behavior suitable for use with children on the cusp of entering the school system. To this end, children were instructed to build a tall tower (*height constraint*) that was striped—i.e., alternating between colors (*striped constraint*)—with Duplo blocks. In this particular study, we aimed to evaluate this task cross-sectionally in 2-year-olds and investigate whether performance on this task is related to performance on standardized measures of executive functions. It was hypothesized that planning actions according to multiple goal constraints would improve over the 3rd year of life and that children's planning ability would be related to their performance on standard tests of the core components of executive functions. If true, then the quality of tower building could be used as a proxy measure of executive function development.

## Method

### Participants

A total of 70 2- to 3-year-olds participated in this study. The sample consisted of 20 24-month-olds (*M* = 24.77 months, *SD* = 12.69 days, 8 females), 20 30-month-olds (*M* = 30.10 months, *SD* = 21.23 days, 12 females), and 30 36-month-olds (*M* = 36.50 months, *SD* = 19.20 days, 18 females). This study was part of a larger study into different types of action sequence planning development in early childhood. These children's performance on some more standard EF tasks (i.e., inhibitory control, task switching, and working memory) has been reported elsewhere (Schröer et al., 2022). Participants were typically developing children with no reported color blindness recruited from a database of interested parents. Families were contacted by phone or email and were informed about the test procedures prior to giving informed consent. Participation was voluntary and families received reimbursement of travel expenses, a present, and a certificate for participation.

As a result of COVID-19 restrictions, all 36-month-olds were tested with all adults wearing a face covering and increased distance between the experimenter and the participant. A total of 13 participants were excluded from the data analysis because (i) the child did not follow any instructions (*n* = 1), (ii) the caregiver influenced the testing session (*n* = 2), (iii) the child had no data for the working memory task (*n* = 1), (iv) the child had no data for the Duplo task (*n* = 1), or (v) the child did not pass the initial requirement of building a tower of more than 2 blocks high (*n* = 8, see planning task procedure).

### Procedure

Participants were tested in a quiet room with their caregivers seated behind them. The children were seated in an age-appropriate chair at a table. They were presented with the tasks in the order described below. The working memory, motor competence, and inhibition tasks were also reported by Schröer et al. (2022). A set-shifting task for 2-year-olds (Trucks game; Hughes and Ensor, 2006) was also performed but was shown to be insensitive to age and excluded from the analysis (Schröer et al., 2022). All procedures were approved by the local ethics committee and conducted according to the Declaration of Helsinki.

#### Planning task

**Figure 2:**
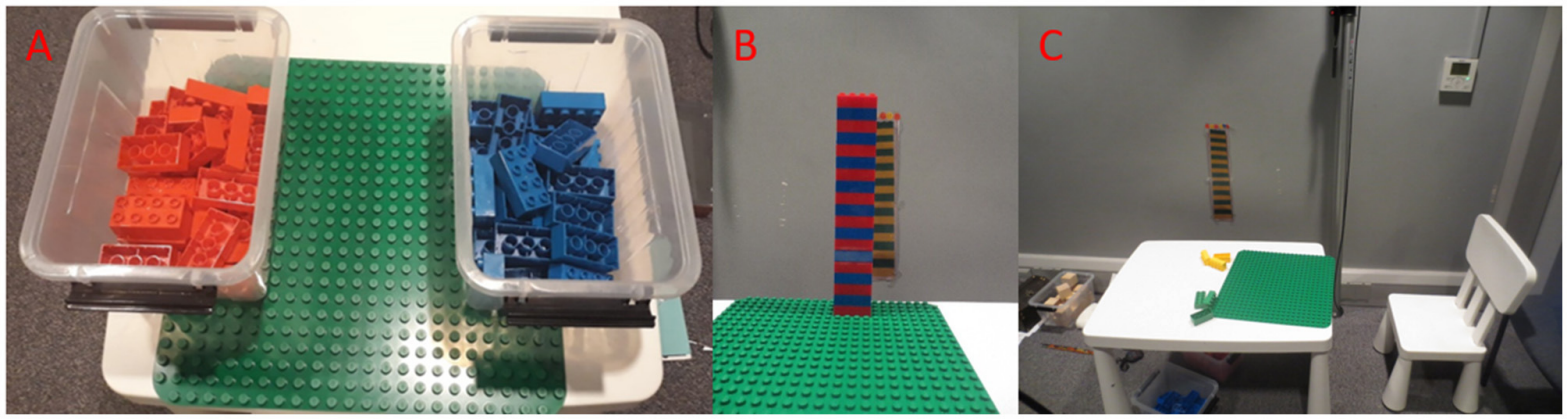
Set-up of the tower task. **(A)** the child's workspace, **(B)** a completed tower with a reference picture in the back, and **(C)** the whole workspace with the reference picture.

Children were instructed to build a tower out of Duplo blocks for a bunny puppet named *Fluffy*. Children had to take into account two constraints: (1) *height constraint:* making the tower as tall as the tower displayed on the wall (20 blocks, 40 cm) and (2) *striped constraint:* making it striped by alternating between two different sets of colored blocks. Fluffy showed how the child should make a striped tower using three yellow and three green blocks similar to the picture of the tower fixed to the wall next to their table. The picture was present to help explain the task constraints and served as a goal reminder. Both task constraints were explained explicitly to each child. Children were presented with one box of blue blocks, one box of red blocks, and a green building plate (Figure 2) and were given one attempt to build the tower.

The task was considered complete when the child indicated that their tower was finished. Children's performance was coded offline. The height constraint was coded as the height of the tower in the number of blocks. This variable was transformed into a binary variable reflecting the success on the height constraint: 1 for a successful tower between 15 and 25 blocks (i.e., between 30 and 50 cm in height; chosen in advance of running the analysis) and 0 for a smaller or taller tower. The ability to successfully complete the striped constraint was coded as the number of color switches on the tower divided by the total number of blocks minus 1. This resulted in a proportion score between 0 and 1, with 0 indicating only one color used and 1 indicating a perfect striped tower. As above, eight participants were excluded from the analysis because they failed to build a tower of more than two blocks high. This criterion ensured that each child included had an understanding of the goal.

Because of the limited verbal abilities of children in this age range (Colson and Dworkin, 1997), participants were asked to confirm whether they believed they had achieved the task goal by referencing a picture of a similar target Duplo tower located next to their workspace throughout the task. Most children confirmed non-verbally by nodding that they believed their tower was equal to the picture. Furthermore, only children who acted in line with the goal (i.e., who placed at least 3 blocks in a tower) were included in the analysis, ensuring that only children who understood the task instructions were included.

#### Working memory task

Working memory updating (WM) was assessed using a spinning pot task (Hughes and Ensor, 2005). Six stickers were hidden in eight distinct boxes arranged on a spinning Lazy Susan tray, and the child had to find all stickers while opening as few boxes as possible. The tray was spun after opening each box. Children could reopen a box that had been opened previously. The score was calculated as 16 (maximum number of boxes opened) minus the number of errors. The number of errors was calculated as the number of boxes opened minus the number of stickers.

#### Inhibition task

Inhibition skills were assessed with the magic wand task (Friedman et al., 2011). Children were instructed not to touch a glitter wand right in front of them for 30 s, while the experimenter looked away. The data were transformed into a binary variable as children either grasped the wand within the first 7 s or waited for the entire 30 s: 0 reflected children who touched the wand, while 1 reflected children who waited for the entire 30 s.

## Results

All analyses were carried out using RStudio (version 1.2.1335). The data demonstrating that performance on both the working memory task as well as the inhibition task improves with age in this sample can be found in Schröer et al. (2022). There were no significant differences in the age of children who were excluded from the sample as compared to those who remained in the analyses for both the 24- and 30-month-old groups (24 months: *t*(17) = 1.10, *p* =.332; 30 months: *t*(17) = 0.87, *p* =.398). We first discuss performance on the tower building task before turning to an examination of the association between performance on that task and traditional EF measures.

### Age-related effects on planning

#### Tower height constraint

**Figure 3:**
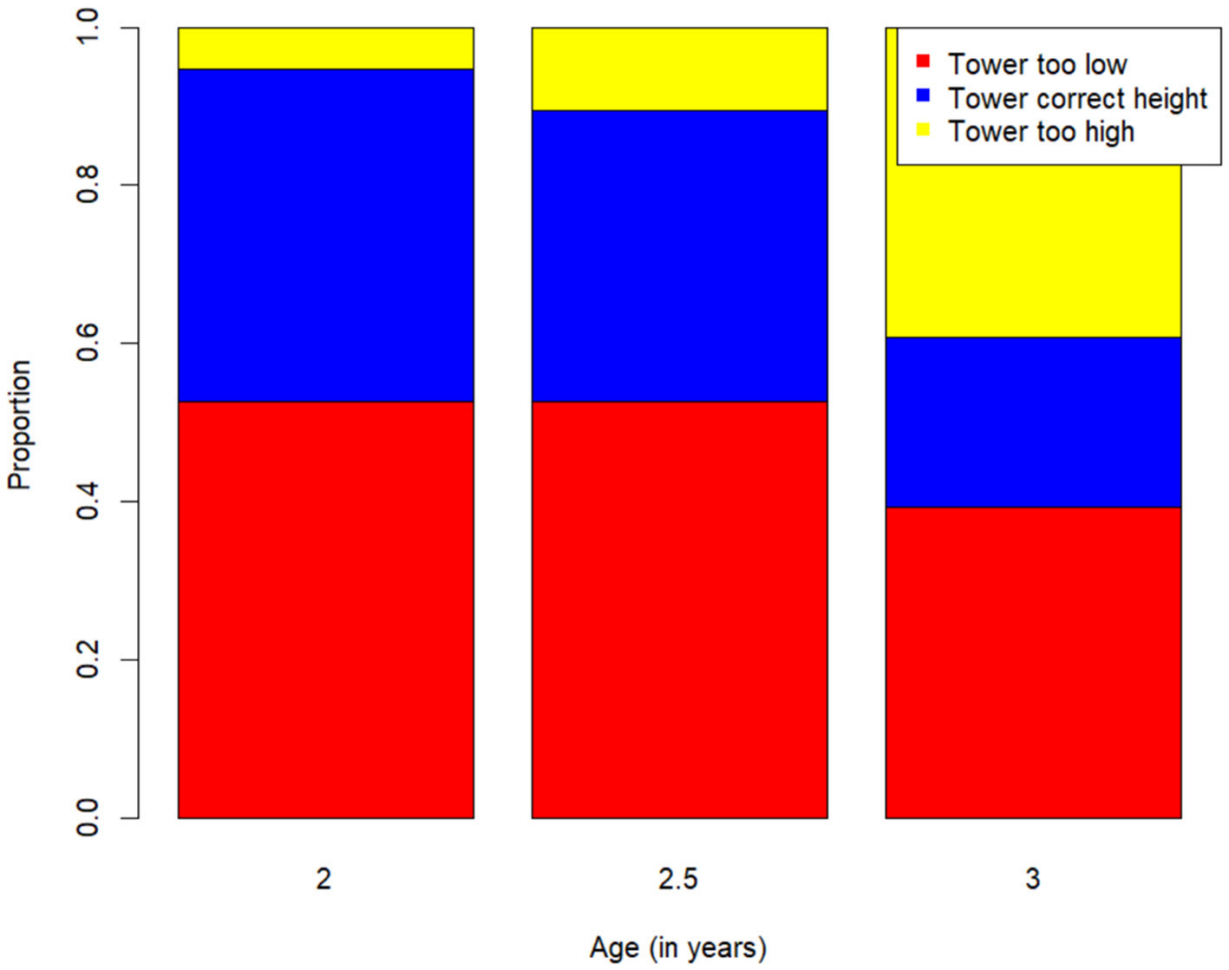
Proportion of children for each age group who successfully completed the tower (blue), built the tower too low (red), or built the tower too high (yellow).

Figure 3 shows the proportion of participants in each age group who built the tower to the correct height (between 15 and 25 blocks; blue), built it too low (red), or built it too high (yellow). A chi-squared test of association showed no effect of age group on the ability to achieve the constraint of height (χ^2^(2) = 5.12, *p* = .063).

#### Tower striped constraint

**Figure 4:**
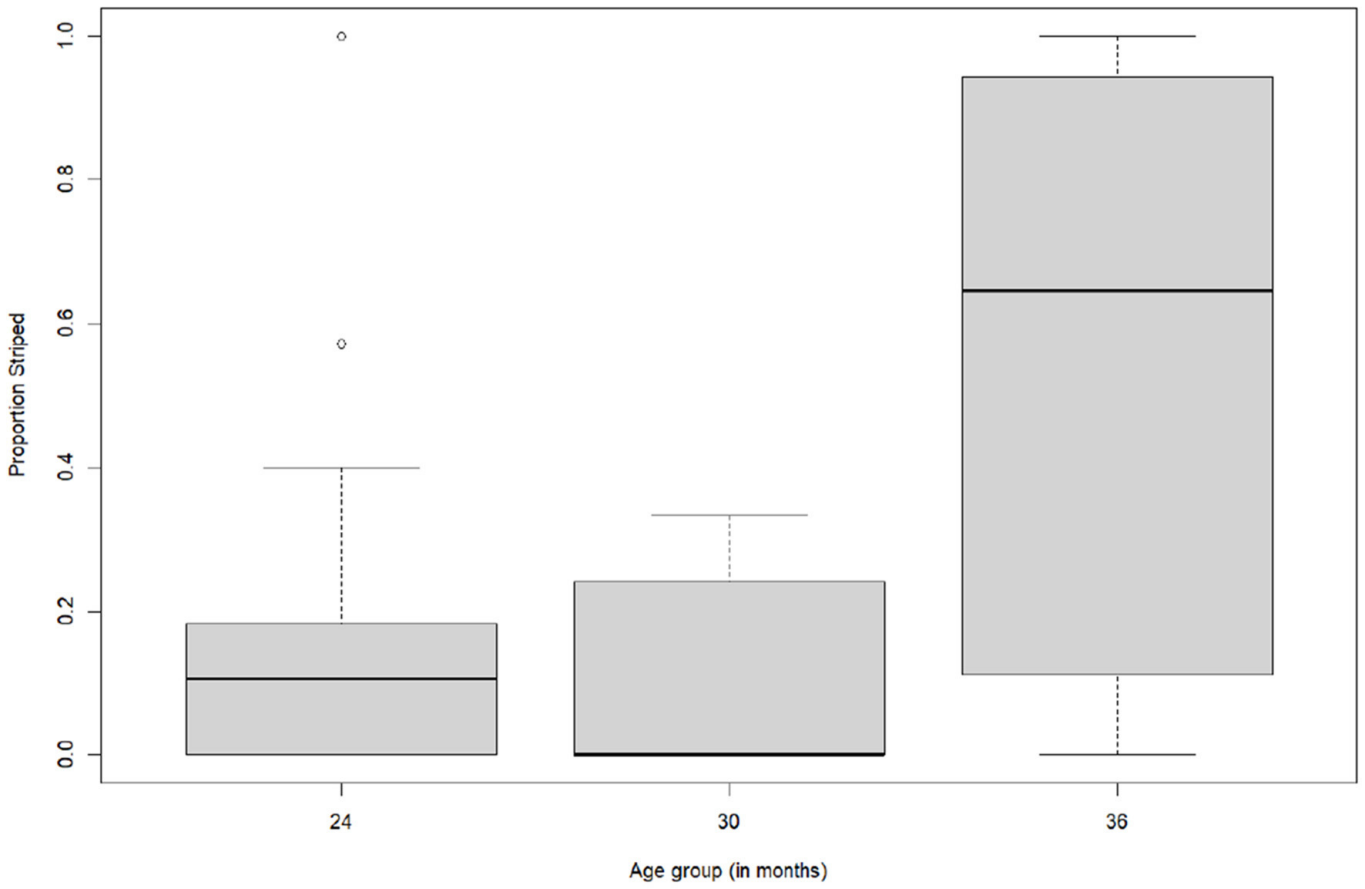
Boxplots for the proportion striped (constraint of striped) per age group.

A stepwise linear regression with age group as a predictor and the proportion striped score as a dependent variable showed that age group was a significant predictor of proportion striped (*F* (1.56) = 15.03, *p* < .001, Table 1). Older toddlers were more successful at adhering to the striped constraint (Figure 4).

**Table 1:** Stepwise linear regression model with proportion striped (striped constraint) predicted by age group.

	***B***	***SE***	***B***	***t***	***P***
**Step 1: Proportion striped**					< .001
Intercept	−0.76	0.29		−2.63	.011
Age group	0.04	0.01	0.46	3.88	< .001
*R^2^* = .212					

#### Relation between striped and height constraint

**Figure 5:**
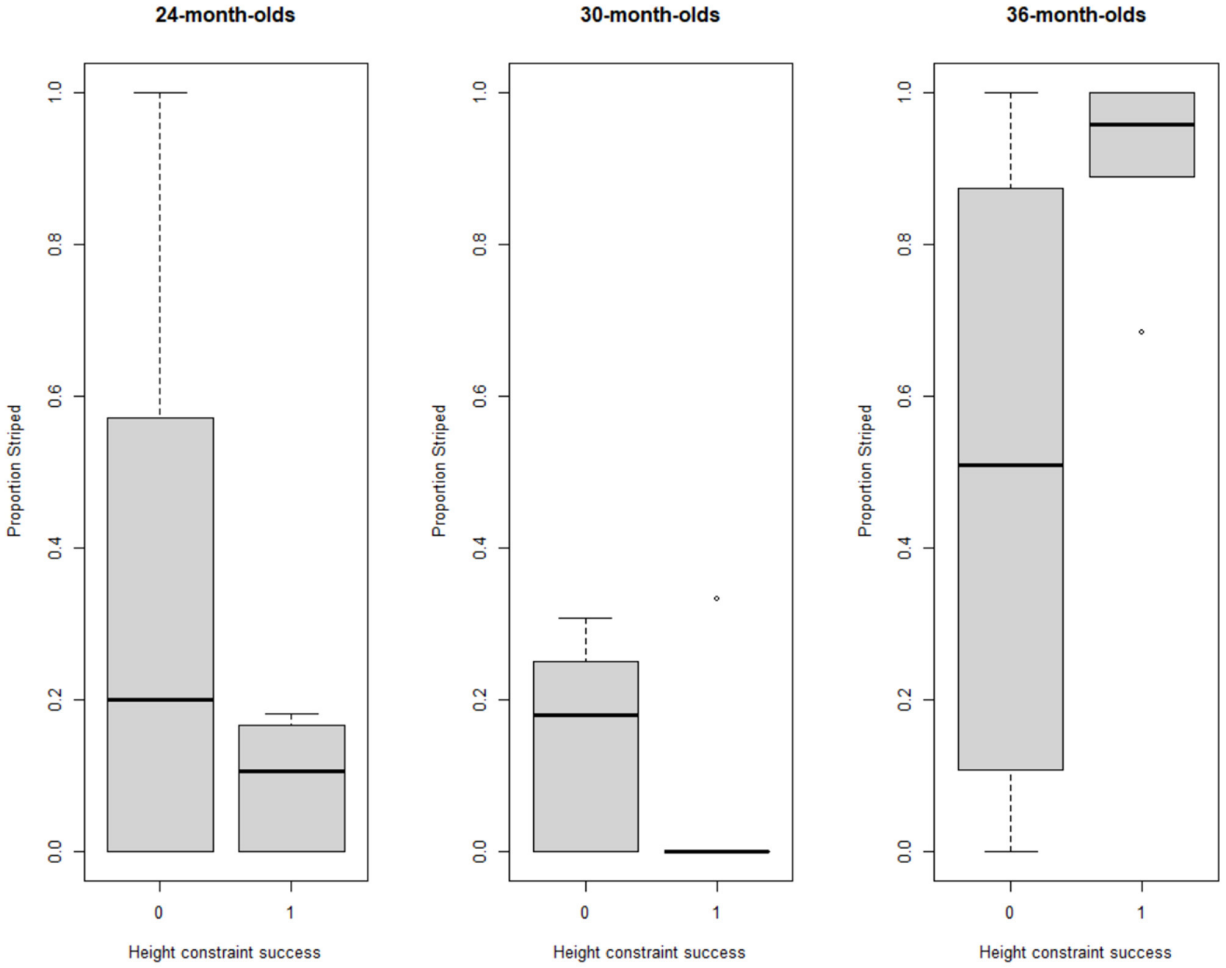
Boxplots for the relation between proportion striped (striped constraint) and success on height constraint (1 = tower to correct height, 0 = tower incorrect height) per age group.

A binary logistic regression was used to investigate whether performance on the striped constraint predicted success on the height constraint. At the group level, performance on the striped constraint was not related to performance on the height constraint (χ^2^(1) = 0.28, *p* = .597). However, looking at each age group separately, there was no significant relation in 24-month-olds (χ^2^(1) = 2.82, *p* = .093), in 30-month-olds (χ^2^(1) = 2.61, *p* = .107), or in 36-month-olds, the proportion striped was a significant predictor of the success on the height constraint (χ^2^(1) = 8.31, *p* = .004). In other words, for 36-month-olds, children who succeeded on the constraint of height were also likely to have achieved a high score on the striped constraint (Figure 5).

### Relation between task execution and EF measures

#### Inhibition

**Figure 6:**
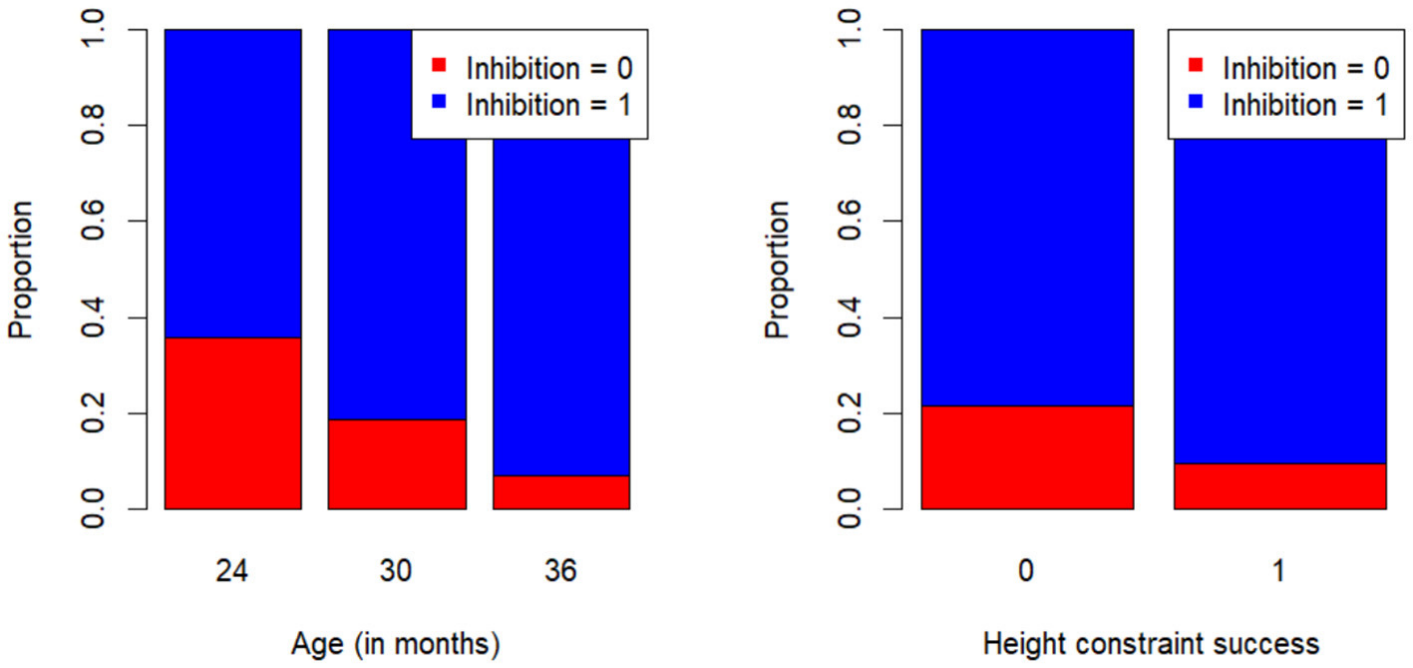
The proportion of children who succeeded on or failed the inhibition task as a function of age group (left panel) and the height constraint (right panel). Note: Children who succeeded in the inhibition task scored 1, while children who failed scored 0. Similarly, children who built the tower to the correct height scored 1 on the height constraint success score, while children who failed to build the tower to the correct height scored 0.

A stepwise binary logistic regression with age group, working memory score, height constraint success, and proportion striped (striped constraint) as predictors and the inhibition score as a dependent variable showed that height constraint success predicted the inhibition score over and above age group (only age group: χ^2^(1) = 4.98, *p* = .026, *Nagelkerke R*^2^= .138; age group and height constraint: χ^2^(1) = 4.43, *p* = .035, *Nagelkerke R*^2^= .251). As shown in Figure 6, older children and children who built the tower to the correct height were more likely to succeed on the inhibition task (and score 1). The striped constraint did not significantly predict the inhibition score. Working memory did neither significantly add to the model nor separately predict inhibition (χ^2^(1) = 0.20, *p* = .657).

#### Working memory

**Figure 7:**
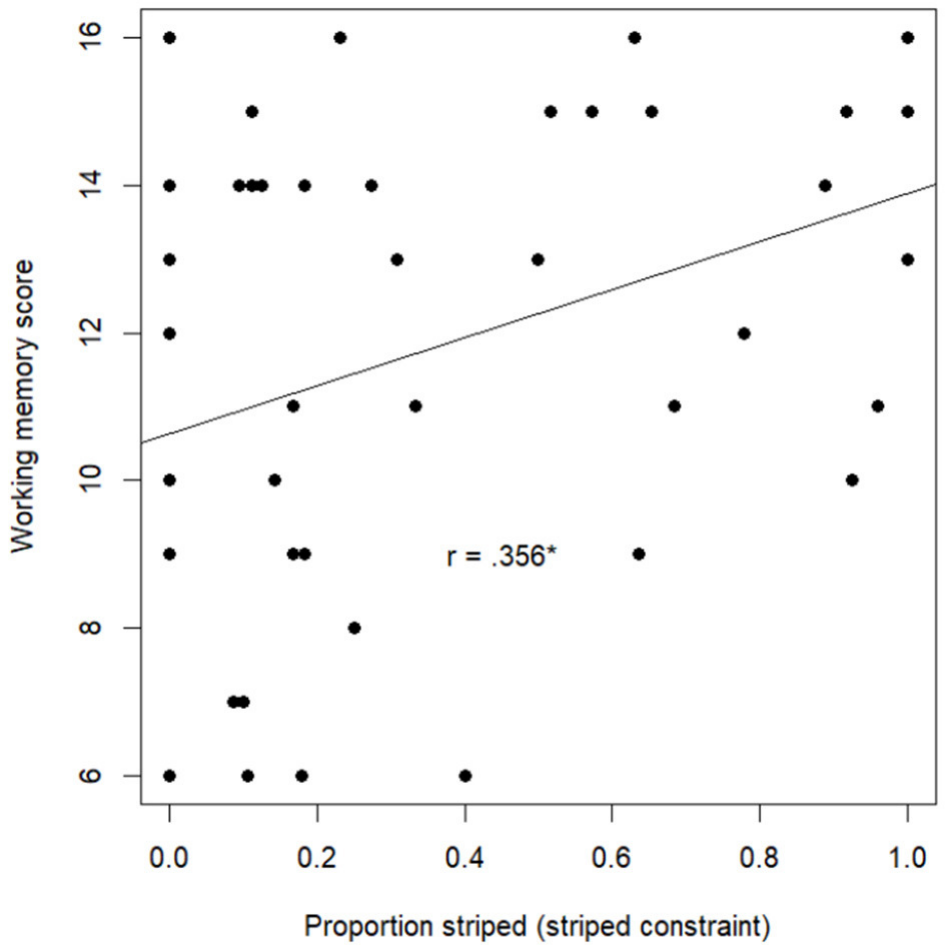
Relationship between proportion striped (secondary constraint) and working memory score.

A stepwise linear regression with age group, inhibition score, height constraint success, and proportion striped (striped constraint) as predictors and working memory score as dependent variable showed that proportion striped (striped constraint) significantly predicted working memory score (*F*(1.55) = 7.96, *p* = .007, Table 2). A higher proportion striped was associated with a higher working memory score (Figure 7). The height constraint was not significantly related to working memory. Inhibition scores did not significantly improve the model.

**Table 2:** Stepwise linear regression model with working memory score predicted by a striped constraint.

	***B***	***SE***	***B***	***t***	***P***
**Step 1: Working memory**					.007
Intercept	10.64	0.58		18.37	< .001
Proportion striped	3.26	1.15	0.36	2.82	.007
*R^2^* = .126					

## Discussion

This study presents a simple and cost-effective way of assessing planning and executive functions in very young children in naturalistic environments. Children participated in a task in which they were instructed to build a Duplo tower taking into account two goal constraints: the height and the striped constraint. The task is simple and naturalistic, allowing its use in nurseries and school settings and making it particularly well suited for use in future to investigate the relationship between planning and school readiness. It is a coarse measure of EF level of development which can be used as a screening instrument. One advantage of the Duplo tower task is that it does not require a language response. It is also simple to operationalize with materials commonly available in nurseries or at home. Most 2-year-olds showed evidence of acting in line with the goal, that is, they built a tower of at least three blocks, demonstrating that they at least understood the instructions and were motivated to act in line with the instructions. Children between 2 and 3 years of age were equally able to achieve the height constraint; however, the ability to achieve the striped constraint improved with age. Older children were more likely to coordinate the two constraints effectively and build a striped tower.

Importantly, inhibition scores measured using a standard task could be related to performance on the height constraint. Children who were successful on the height constraint were also more likely to be better at the inhibition task. Moreover, working memory was predicted by performance on the striped constraint, and better performance on the planning task was associated with a higher working memory score. Thus, while the tower task does not have the resolution of many of the standard EF tasks, it provides an easy-to-use, fun, and natural screening tool for EF-related school readiness factors, such as planning, working memory, and inhibition. This might be especially important given that, in late adolescence, planning is known to be an important factor related to academic success (Baars et al., 2015), and many studies have already demonstrated a relationship between working memory and inhibition and between school readiness and school success in childhood (Bull and Scerif, 2001; Blair and Raza, 2007; Bull et al., 2008; Gathercole et al., 2008; Clark et al., 2010; Espy et al., 2010; Welsh et al., 2010; Monette et al., 2011; Fitzpatrick and Pagani, 2012; Shaul and Schwartz, 2013).

Our results are also consistent with the hypothesis that the development of executive functions and complex action planning are related (McCormack and Atance, 2011), specifically that planning is a higher-level EF that draws upon the lower-level core EF components (Diamond, 2013). First, working memory could be predicted by planning action sequences (i.e., striped constraint). This is consistent with a previous study in which working memory was related to planning simple alternating actions in toddlerhood (Schröer et al., 2022). Second, inhibition was predicted by age as well as the height constraint in the action sequence planning task. In a previous study, inhibition was also found to be related to avoiding actions irrelevant to the goal hierarchy in the preschool period (Schröer et al., 2021). Thus, it might be that inhibition is essential in avoiding actions that do not achieve the goal and focusing on actions that accomplish the goal. In short, our results provide further evidence that planning is dependent on lower-level EF components such as working memory and inhibition (Diamond, 2013).

Interestingly, the working memory score was not predicted by the height constraint, while inhibition was not predicted by the striped constraint in the Duplo tower task. Furthermore, working memory did not predict the inhibition score. In adulthood, the three core components of EF are correlated but functionally separated (Miyake and Friedman, 2012). This distinction into separate but related core aspects of EF is not clear in infancy (Hendry et al., 2016), but several studies have detected separable components in 2- to 3-year-old children (Garon et al., 2014; Skogan et al., 2016). In the current study, working memory and inhibition scores were not associated, suggesting separable functions. Similarly, Gottwald et al. (2016) did not find a correlation between simple inhibition and working memory in 18-month-olds. Thus, our study suggests that both working memory and inhibition contribute independently to the higher-level EF of planning. Further research could investigate this link between the three lower EF components and how these components relate to higher-level EFs such as planning, reasoning, and problem-solving.

One limitation of the current study is that a cross-sectional design was used to investigate age-related effects on the Duplo planning task. Future studies could investigate the improvement of planning over preschool age by using a longitudinal design to examine whether this task can be used to predict EF and school readiness prospectively. A second potential limitation is that we did not take into account the children's height. All children were seated at the same table and the same chair, and the picture was located at exactly the same location on the wall. However, as there were no age effects in the height construction constraints, it is unlikely that age-related physical height of the children influenced their tower building performance.

Future studies could also use this Duplo tower task to assess the relationship of planning (higher-level EF) in very early childhood with school readiness and academic success in later childhood. This research area is of special interest since many studies have assessed EFs to relate to school readiness and success among children of 4–5 years of age (e.g., Bull et al., 2008; Clark et al., 2010; Welsh et al., 2010; Monette et al., 2011), while, in many countries, the first formal school systems begin during 3 to 4 years of age. For example, prior research has shown that working memory in toddlerhood is related to better classroom engagement and school success in mathematics and literacy in kindergarten (Fitzpatrick and Pagani, 2012), suggesting that assessing EF abilities in toddlerhood is important to understand the relationship between EFs skills and school readiness and school success later.

The aim of the current study was to develop a naturalistic measure of planning that could be used in the real world. A key challenge in psychology and neuroscience is to move away from traditional laboratory studies and investigate the development and brain function in the real world (Dahl, 2017; Pinti et al., 2018; Matusz et al., 2019). Studying development in controlled lab contexts removes the richness of the real world (Cantlon, 2020). The key challenge of moving away from lab-based tasks to naturalistic and easily administered behavioral tasks is also present in educational neuroscience (Janssen et al., 2021). A first step in addressing this issue is to create easily administered behavioral tasks that can be used in a nursery or school setting to assess cognitive and neurocognitive processes such as planning or other executive functions that are relevant for understanding the relationship between cognition and school readiness and school success. The Duplo tower task is a good example of this; it is an easily administrated naturalistic behavioral task that can assess very young children's planning, working memory, and inhibition skills. Furthermore, it is simple and does not require any complex material meaning that it could be used to assess these abilities in everyday nursery or school settings. These simple naturalistic behavioral tasks could be used in future to assess the relationship between cognition and education in nurseries, schools, or anywhere outside of the traditional laboratory, for example, the relation between planning abilities in toddlers and school readiness in the nursery environment.

The next step for educational neuroscience is to adopt simple naturalistic tasks such as the Duplo tower task to assess brain development using wireless neuroimaging methods. This would allow the investigation of the functional brain development associated with EF development that is critical for school readiness. In cognitive neuroscience, research into real-world neuroscience has already demonstrated that wireless fNIRS can be used to examine brain patterns during real-world behavior in adults (Pinti et al., 2015; Balardin et al., 2017), making it a particularly interesting method to apply to educational neuroscience with naturalistic tasks (Janssen et al., 2021). Similarly, mobile EEG can be used to assess differences in processes in the classroom, such as attention (Xu et al., 2022). In the same way, as adult cognitive neuroscience must be taken out of the laboratory and into the real world, educational neuroscience research needs to be taken out of the laboratory and into real classrooms.

## Data availability statement

The raw data supporting the conclusions of this article will be made available by the authors, without undue reservation.

## Ethics statement

The studies involving human participants were reviewed and approved by Birkbeck College Ethics Committee. Written informed consent to participate in this study was provided by the participants' legal guardian/next of kin.

## Author contributions

DM, RC, and LS conceived and designed the study and wrote the manuscript. LS collected the data. DM and LS analysed the data. All authors contributed to the article and approved the submitted version.
